# Effect of Spent Mushroom Compost of *Pleurotus eous* Strain P-31 on Growth Performance and Nodulation of Cowpea (*Vigna unguiculata* Walp.)

**DOI:** 10.21315/tlsr2022.33.3.8

**Published:** 2022-09-30

**Authors:** Wiafe-Kwagyan Michael, Odamtten George Tawia, Kortei Nii Korley

**Affiliations:** 1 Department of Plant and Environmental Biology, University of Ghana, P.O. Box 55, Legon, Accra, Ghana; 2 Department of Nutrition and Dietetics, School of Allied Health Sciences, University of Health and Allied Sciences, PMB 31, Ho, Ghana

**Keywords:** Sandy Loam Soil, Cowpea, Rhizobial Population, SMC, *Pleurotus eous* P-31, Nodulation, Nodule Index

## Abstract

This study investigated the influence of spent mushroom compost (SMC) of *Pleurotus eous* strain P-31 on the growth, development and soil rhizobial population associated with nodulation of cowpea (*Vigna unguiculata* Walp.) black-eye variety, under greenhouse conditions at 28 ± 2ºC for 12 weeks. Sandy loam soil was combined with different percentages of SMC to obtain the following combinations (0%, 5%, 10%, 15%, 20%, 25%, 30%, 100%). Lower concentrations, SMC (5%–25%) promoted plant height, number of leaves, total leaf area, total chlorophyll, chlorophyll a and b as well as dry matter accumulation of shoot and roots after 12 weeks at 28°C–32°C. Soil: SMC concentrations beyond 30% SMC variably depressed the various developmental criteria used in assessing growth. The trend obtained in the assessed parameter were statistically significant (*p* ≤ 0.05) in decreasing order: 5% SMC < 10% SMC < 15% SMC, < 20% SMC, < 25% SMC, < 30% SMC, < 100% SMC. The cowpea plant efficiently assimilated nitrogen (N_2_) from the soil: compost. Nodule formation by cowpea was commensurate with increasing percentage of spent compost was highest in 5% SMC (89/plant) and declined with increasing proportion of SMC: soil mixture up to 25% but nodulation of cowpea plant was completely depressed in the absence of soil (100% SMC) pots. The Nodule Index data showed that the best nodule size and weight were formed by cowpea growing in medium containing 5% SMC (18) and 10% SMC (12) and thereafter declined. The nodules were red to pinkish in colour epitomising leghaemoglobin which could initiate nodulation and N_2_ fixation in soil. This study has shown that 5% SMC–20% SMC could provide favourable conditions in soil as a biofertiliser to improve the growth, development and nodulation of cowpea.

**Highlights**
- Sandy loam soil was amended with spent mushroom compost of *Pleurotus eous* to assess its effects on the development and nodulation of cowpea (*Vigna unguiculata*).
- Lower SMC (5%–25%) promoted vegetative growth (i.e., promoted plant height, number of leaves, total leaf area, total chlorophyll, chlorophyll a and b) and nodulation of cowpea.
- The Nodule Index data showed that the best nodule size and weight were formed by cowpea growing in medium containing 5% SMC (18) and 10% SMC (12) and thereafter declined whereas no nodules were formed by cowpea grown in the absence of soil (i.e., SMC only or 100% SMC).

## INTRODUCTION

The expansion of the mushroom industry is an even global phenomenon; accentuated by their increased campaign on health, nutritional and medicinal benefits (Aderemi *et al*. 2014). In recent years, efforts have also been made to recycle agro-industrial wastes to serve as biofertilisers to increase agriculture production to feed the global exponential increase in human population. Agriculture and the food industries have aggravated the current environmental pollution, by its added solid waste, wastewater or gaseous pollution.

Generally, mushrooms are produced on natural agro-lignocellulose such as hay, straw, horse bedding, poultry litter, corn cobs, corn stover, cotton seed musk, coffee residues (hulks, stalks and leaves, cassava peels, cocoa pod husk/shells, banana fronds, agave waste, soy pulp, and many more not exempting rice husk, rice waste etc.) (Wiafe-Kwagyan *et al*. 2016; Adedokun & Akuma 2014; Aderemi *et al*. 2014; Obodai *et al*. 2003). More than 10 million metric tonnes of spent mushroom compost is expected to be generated annually worldwide (Wiafe-Kwagyan *et al*. 2016; Aderemi *et al*. 2014; Gonani *et al*. 2011; Philippoussis *et al*. 2004; Lau *et al*. 2003). It has now become a subject of great importance, especially in developing countries where there are formidable challenges for effective waste disposal (Gonani *et al*. 2011; Philippoussis *et al*. 2004; Wiafe-Kwagyan & Odamtten 2018).

Although spent mushroom compost (SMC) is often regarded as an agricultural waste with little inherent value, yet there is still value in the SMC as it has been shown to be rich in nutrients and organic matter that can provide benefits to other agricultural and non-agricultural sectors. After suitable pre-treatment, spent mushroom compost can completely or partially substitute the growing media for the cultivation of different economically important horticultural crops (Roy *et al*. 2015). Spent mushroom compost has been shown to be a food source of carbon, nitrogen and other essential elements and the nitrogen content varies from 0.4%–13.7% with a C:N ratio of 9 to 15:1 which enhances the growth of plants (Roy *et al*. 2015).

Spent mushroom compost can also be used as feeding material for vermicomposting, plant disease management, and preparation of organic mineral biofertiliser and also for bioremediation of contaminated soils (Ahlawat *et al*. 2010; Uzun 2004). Although, there are other alternative uses of SMC, these are warrant supporting scientific data particularly in developing countries such as Ghana and this lack of scientific data on application of SMC for maximum benefits have not been extremely exploited (Quartey & Chýlková 2012). Recently, Wiafe-Kwagyan and Odamtten (2018) have shown that the use of *Pleurotus eous* strain P-31 spent mushroom compost as a biofertiliser provided favourable soil conditions for the good growth and yield of tomato (*Solanum lycopersicum* Fr. *Lycopersicum esculentum*) and pepper (*Capsicum annum* L.) in Ghana under screen house conditions. However, unscientific ways of using SMC can also create several problems in soil including the accumulation of salts, leading to harmful effects on some crop plants. The use of SMC as biofertiliser cannot be generalised and therefore a case for this study to ascertain to assess their usefulness in crop production to support their universal use in agriculture. To date, high cost of chemical fertiliser to developing countries not excepting the concern for sustainable soil productivity, and ecological stability in relation to the chemical fertiliser use, has emerged as important issue for policy maker (Shahnawaz *et al*. 2009). There is also the renewed attendance to the use of organic manures from farmlands, compost and green manure as source of plant nutrients (Roy *et al*. 2015; Marques *et al*. 2014; Uzan 2004; Çayci *et al*. 1998; Singh *et al*. 1988). There are two schools of thought by researchers on this issue. Some workers observed that organic manure and biofertiliser improved availability of some minerals in the soil especially the transfer of nutrients from land to crop plant. Others also indicate that these minerals negatively influence crop yield and affected chlorophyll content and other growth parameters. Spent substrates from same or different types of mushrooms may vary in their release of nutrients to different growing plants in soil with SMC serving as biofertiliser. Although SMC from *P. eous* P-31 cultivation on rice waste provided favourable soil conditions as a biofertiliser for growing pepper and tomato in Ghana (Wiafe-Kwagyan & Odamtten 2018), farmers in different parts of Ghana are not conversant with the use of SMC as manure or biofertiliser for soil conditioning for various field crops because of paucity of scientific data to back its use on commercial scale.

Cowpea (*Vigna unguiculata* Walp.) is an important legume widely cultivated on commercial and semi-commercial scales in almost all farmlands in most parts of Ghana and is a good candidate crop to use as a test plant. The legume has an interesting symbiotic nitrogen fixation process in their root nodules in association with *Rhizobium*, one of the most studied symbiotic nitrogen fixing bacteria found in cowpea root nodules and other leguminous plants. This article reports novel information on the role of spent mushroom compost *P. eous* P-31 used as soil biofertiliser to influence soil rhizobial, population, nodulation, growth and yield of cowpea in amended soil seeded with cowpea seedlings.

## MATERIALS AND METHODS

### Materials

The research was carried out at the Department of Plant and Environmental Biology greenhouse, University of Ghana, Legon. Potted plastic buckets of dimension (22.5 cm long × 16.0 cm in width × 30.5 cm deep). Cowpea seeds used as test plants were collected from the Genetics Unit of the Department of Botany, University of Ghana, Legon.

### Collection of Spent Mushroom Compost and Soil Sample

Spent mushroom compost was collected from the Mycology Unit of Food Research Institute (FRI) under the Council for Scientific into Industrial Research (CSIR). The compost was subjected to at least a minimum of four weeks mycelia spawn run and at most six weeks of cropping period. Loamy soil sample was collected from the University’s farm at the Legon Botanical Garden.

### Experimental Design

The method used by Wiafe-Kwagyan and Odamtten (2018) was employed. The test plant was Cowpea (*V. unguiculata* Walp.) this was chosen because of its prolific cultivation in backyard gardens and on commercial farms in most district and regions in Ghana. To study the effect of the spent mushroom compost (SMC) on vegetative growth of cowpea, a modified method of Polat *et al*. (2009), Gonani *et al*. (2011) and Çaycı *et al*. (1998) was used. The SMC was first sun dried for five days in order to reduce its moisture content and eliminate undesirable resident bio-deterioration agents such as maggot, insects, etc. Subsequently the dried SMC was added to sandy loamy soil (Haatso series) in order to obtain the different combinations of soil and SMC in following percentages; 0% SMC (100% soil. i.e., control growing medium); 5% SMC (5% of SMC to 95% soil); 10% SMC (10% of SMC to 90% soil); 15% SMC (15% of SMC to 85% soil); 20% SMC (20% of SMC to 80% soil); 25% SMC (25% of SMC to 75% soil) and 30% SMC (30% of SMC to 70% soil). SMC in the following ratios 5% SMC, 10% SMC, 15% SMC, 20% SMC, 25% SMC and 30% SMC were added to sandy loam soil to obtain a total weight (w/w) 6.5 kg for each plastic; 100% SMC was used as second control. Thoroughly mixed of SMC and sandy loam soil the growth medium in each plastic bucket was irrigated with 1 L running tap water. Three health seeds were planted into each plastic bucket. After germination, the seedlings of same size and height were selected for the study while the remaining two cowpea seedlings in all treatments were uprooted. The potted cowpea plants in the plastic buckets were arranged in a randomised complete-block design (RCBD) with five replicates for each of the treatments afore-mentioned above. SMC only or soil only medium served as controls for comparison purposes. Seedlings were watered every other day with equal volumes of 500 mL of tap water. The following parameters were recorded on weekly intervals: plant height, number of leaves, floral buds, flowers, fruit, axillary branches, leaf area and chlorophyll content of leaves, stem girth and number of fruits and weight of fruits. Correlation between nitrogen content of growing medium and assimilation of nitrogen by cowpea seedlings in different treatment as well as isolation and identification of *Rhizobium strains* were done after 12 weeks of planting.

### Estimation of Chlorophyll Content of Leaf

Leaf samples from the collected harvested plants were used for the estimation of chlorophyll content after 8 weeks following the procedure outlined by Bansal *et al*. (1999). One hundred milligram (100 mg) fresh leaves were crushed/blended in 20 mL of 80% acetone and the extract centrifuged for 10 min at 1000 rpm. Absorbance of the supernatant was recorded at 645 nm and 663 nm in a Jenway Spectrophotometer (Jenway Model No. 6305 Bacdoworld Ltd. Dunmow, Essex, U.K.). Chlorophyll content (expressed as mg/g) was estimated according to the following formula (Adopted from Bansal *et al*. 1999):


$$\begin{array}{l} \text{Chlorophyll a}=(12.7\times \text{Absorption at }663\;\text{nm})-(2.69\times \text{Absorption at }645\;\text{nm})\;\text{mg}/\text{L}\\ \text{Chlorophyll b}=(22.2\times \text{Absorption at }645\;\text{nm})-(4.67\times \text{Absorption at }663\;\text{nm})\;\text{mg}/\text{L}\\ \text{Total Chlorophyll}=(20.2\times \text{Absorption at }645\;\text{nm})+(8.02\times \text{Absorption at }663\;\text{nm})\;\text{mg}/\text{L} \end{array}$$

### Total Chlorophyll Content of Leaves (CCL)

This was determined weekly after transplanting seedlings using a chlorophyll meter (Optic Science CCM – 200 plus Model, USA) by attaching the chlorophyll meter knob to five randomly selected leaves of equal age and size and the average data was recorded as Chlorophyll Content Index (CCI).

### Number of Leaves and Total Leaf Area

The number of leaves formed was counted weekly for 12 weeks at 28 ± 2°C. Leaf area in mm^2^ was determined by a Digital Area Meter (Model L1-300C, USA) by attaching five randomly selected leaves of same age and size to the instrument.

### Plant Height

This was determined using a meter rule placed firmly on the surface of the substrate to determine the height of the plant in centimeters.

### Dry Matter Accumulation by Plant

After 12 weeks of vegetative growth in the potting buckets, the plants were carefully removed from the soil/compost mixture to avoid breakage and were thoroughly washed in running tap water followed by three changes of clean water in order to remove all the roots as well. The plant was dismembered into shoot and roots, placed in brown envelopes for each treatment before weighing each separately and placing them in an oven (Gallenkamp Oven 300plus Series, England) at 100°C for 24 h and then reweighed after cooling in a desiccator. Method adopted from (Sharma *et al*. 2012).

### Determination of Mineral Content of Soil and Spent Mushroom Compost

The method used by Wiafe-Kwagyan and Odamtten (2018) was employed as a modification of the procedure of Cunniff (1995).

The following elements (N, Ca, Mg, K, P, Cu, Zn, Mn, Pb, Na and Fe) were estimated in the soil and SMC using the conventional methods (Atomic absorption spectrometry, Flame Atomic Emission Spectrometry and Kjeldahl). The total of 250 mg powdered air-dried sample was weighed into a beaker was then placed in ignition muffle furnace (Vectar-furnace, PS3-Sweden) for drying at 400°C for 24 h. Five millitres of hydrochloric acid (HCl) was added to the sample and the solution was dried again; subsequently 5 mL of nitric acid (HNO_3_) was added. After evaporation, the sample was diluted to 50 mL with water. Sodium (Na) and calcium (Ca) content of the ashed sample were determined by flame photometer. K, P, Mg, Cu, Zn, Mn, Fe and Pb were determined by Unicam 929 Atomic Absorption Spectrophotometer (AAS) (Model PinAAcle 900T) (Wiafe-Kwagyan & Odamtten 2018). Nitrogen was estimated using the micro Kjeldahl method (Cunniff 1995).

### Correlation between Nitrogen Content of Growing Medium and Proportion Assimilated by Cowpea or Nitrogen Assimilation from the Growing Medium by Cowpea

The nitrogen content of growing medium before and after planting of cowpea seedlings as well as the nitrogen content of harvested seedlings after 12 weeks of planting were determined using the Kjeldahl method (Association of Official Analytical Chemist [AOAC] 2005).

### Isolation and Identification of *Rhizobium* Strain

This was carried out according to the modified method of Hahn (1966). The root system of cowpea was washed carefully under gentle running water. Nodules on tap root of each plant were cautiously detached for the isolation of the bacterium. The nodules were then put in sterile distilled water containing a little clean acid-washed sand in McCartney tubes and the tubes vigorously shaken to remove gross surface contamination. The nodules were subsequently surface sterilised for 5 min in 0.1% mercuric chloride (3% w/v hydrogen peroxide) and repeatedly rinsed in six changes of sterile distilled water. The nodules were then crushed with a glass rod in few drops of sterilised distilled water in McCartney tubes. Six serial dilutions of 1:10^1^, 1:10^2^, 1:10^3^, 1:10^4^, 1:10^5^, 1:10^6^v/v of the suspensions were prepared. Dilutions of 1:10^4^, 1:10^5^, 1:10^6^v/v were streaked on Petri plates of Congo red-yeast-mannitol agar medium and incubated at 30°C for five days. At the end of incubation period, isolated colonies of the *Rhizobium* species were sub-cultured on Yeast Extract-Mannitol Agar (YEMA) slant in McCartney tubes. The tubes with pure cultures were filed with sterile liquid paraffin to completely submerged the slant and stored in refrigerator at 4°C for further use.

### Influence of SMC and Soil Mixtures on Nodulation of Cowpea and Radicle Development

Sandy-loam soil was amended with SMC with the following rates 5%, 10%, 15%, 20%, 25% and 30% whereas 0% (sandy-loam soil) and 100% (SMC only) served as control treatments, respectively. After the growing medium was thoroughly mixed it was irrigated with 1 L tap water. One cowpea seed was planted 4 cm per bucket 25 cm × 22 cm of height and diameter respectively for all treatments. At the end of 8 weeks of planting the number of Nodule Index (Rebah *et al*. 2002) was used to assess the influence of SMC on nodulation of cowpea seedlings.

The Nodule Index was calculated following the method prescribed by Rebah *et al*. (2002):


Nodule Index=A×B×C≤18

Where A = nodule size, B = nodule colour and C = number of nodules.

**Table:** 

Nodule size	Value	Nodule colour	Value	No. of nodule	Value
Small	1	White	1	Few	1
Medium	2	Pink to red	2	Several	2
Large	3			Many	3

Root nodules were counted and weighed using digital electric weighing balance. Using a sharp scalpel, the nodule was carefully cut open and the colour of the nodules was determined by visual observation of the colour on the cut surface. Using the method adopted by Rebah *et al*. (2002) the Nodule Index was calculated. Nodule’s diameter was measured using a micrometer screw gauge (model flipkart plus mLabs BO1FQGOBY2, UK). Radicle development and nitrogen assimilation was assessed by counting the number of nodules, pods and determining the dry weight accumulation of the roots using oven dry weight method.

### Statistical Data Analysis

Data were analysed by standard ANOVA procedure for randomised complete block design and least significant difference (LSD) at *P* = 0.05 was used. Treatment means were compared using Duncan’s Multiple Range Test, i.e., DMRT Statistical Package for Social Sciences (SPSS) version 16 for Windows.

## RESULTS

### Estimation of Chlorophyll Content of Cowpea Leaves

The spectrophotometric measurement of chlorophyll content (Chlorophyll a, Chlorophyll b and Total Chlorophyll) is summarised in Table 1. Chlorophyll b was highest (20.46 mg/L–23.15 mg/L) in the 0%–5% SMC while Chlorophyll a predominated (12.32–22.57 mg/L) in 10%–25% SMC compost. The compost with 30%–100% SMC compost, Chlorophyll a (11.95 mg/L–18.30 mg/L). Total chlorophyll was highest at 5% – 25% SMC but least at 100% SMC (17.81 mg/L) after 12 weeks.

### Total Chlorophyll Content of Leaves using Chlorophyll Content Index Meter (CCI)

**Figure 1:**
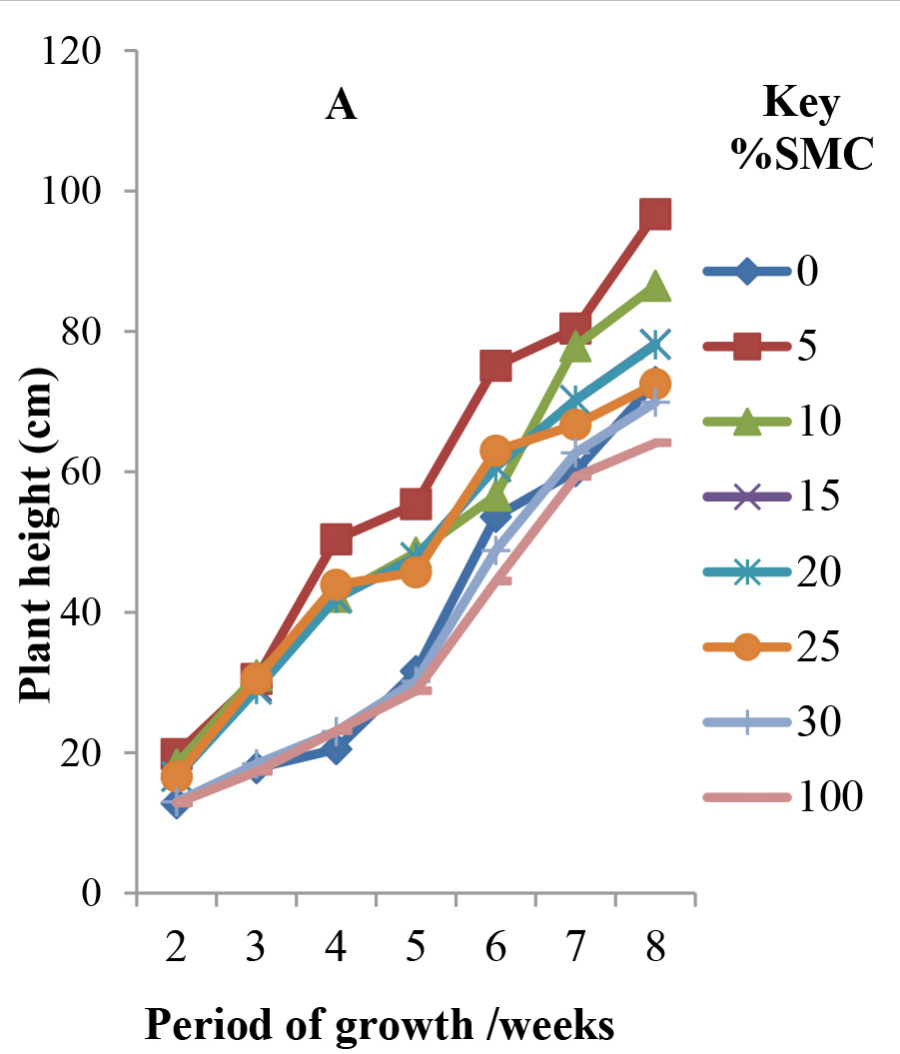

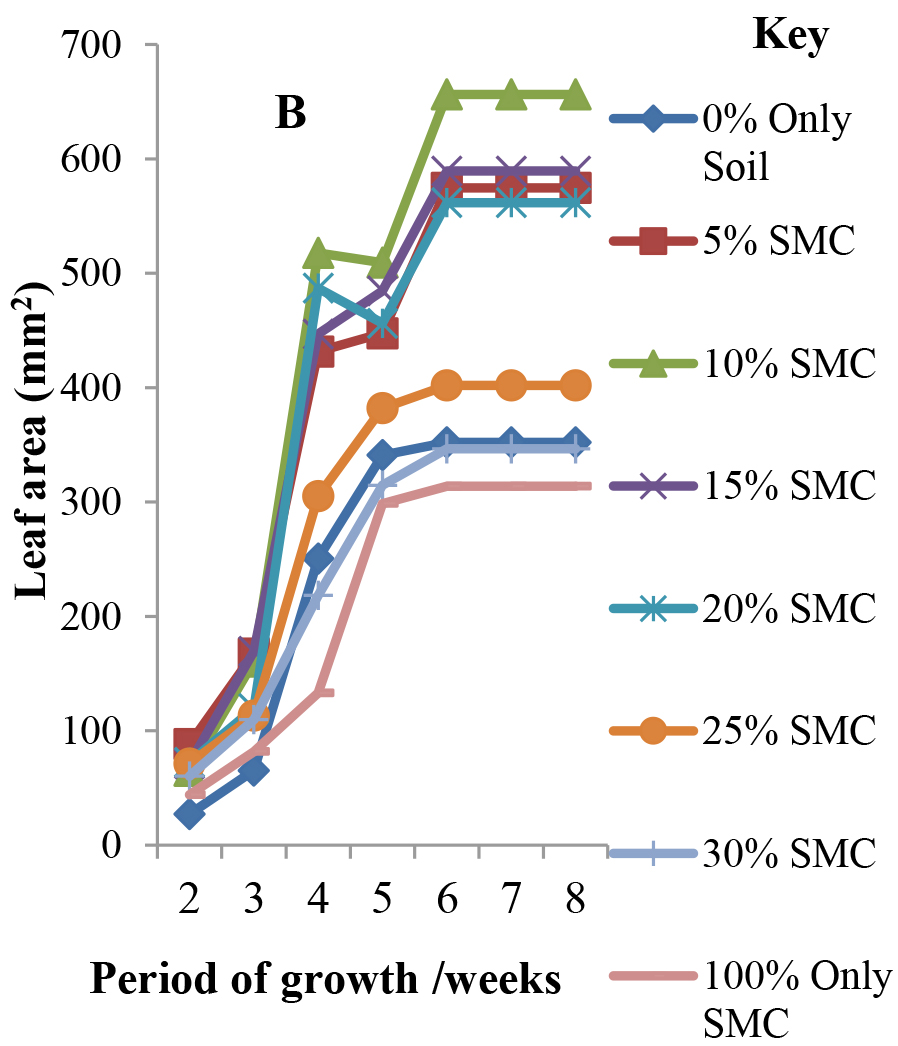

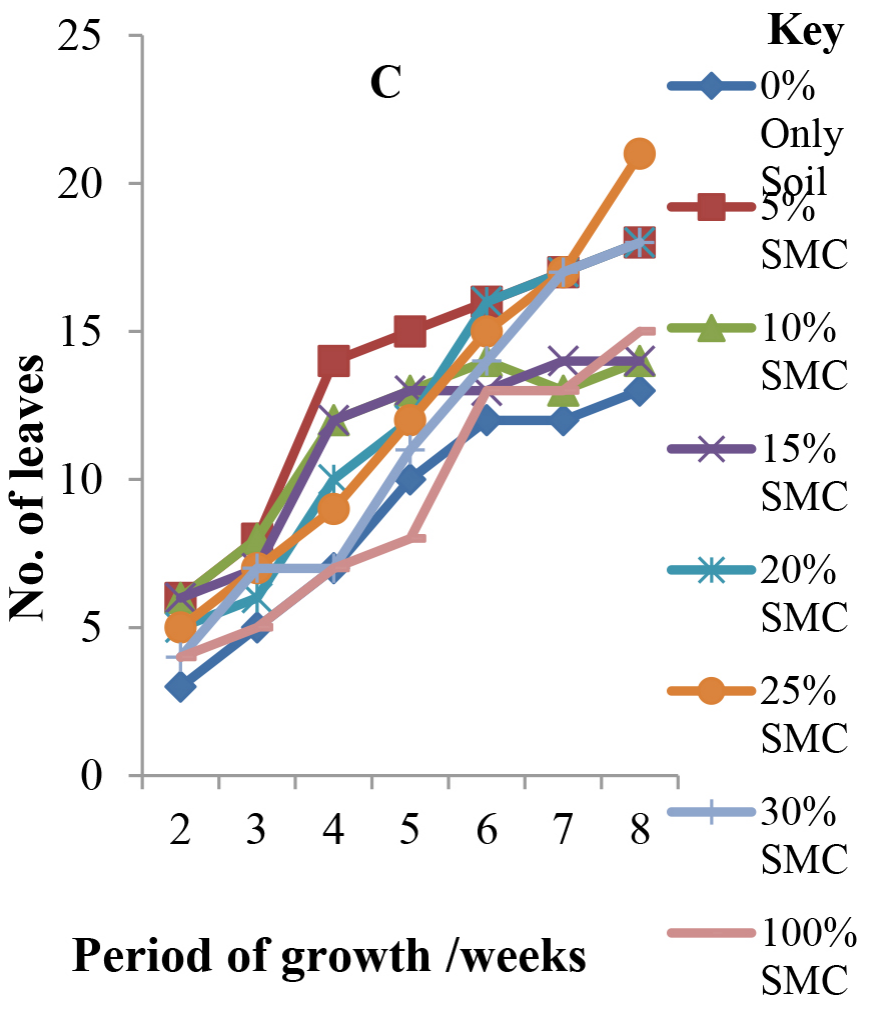

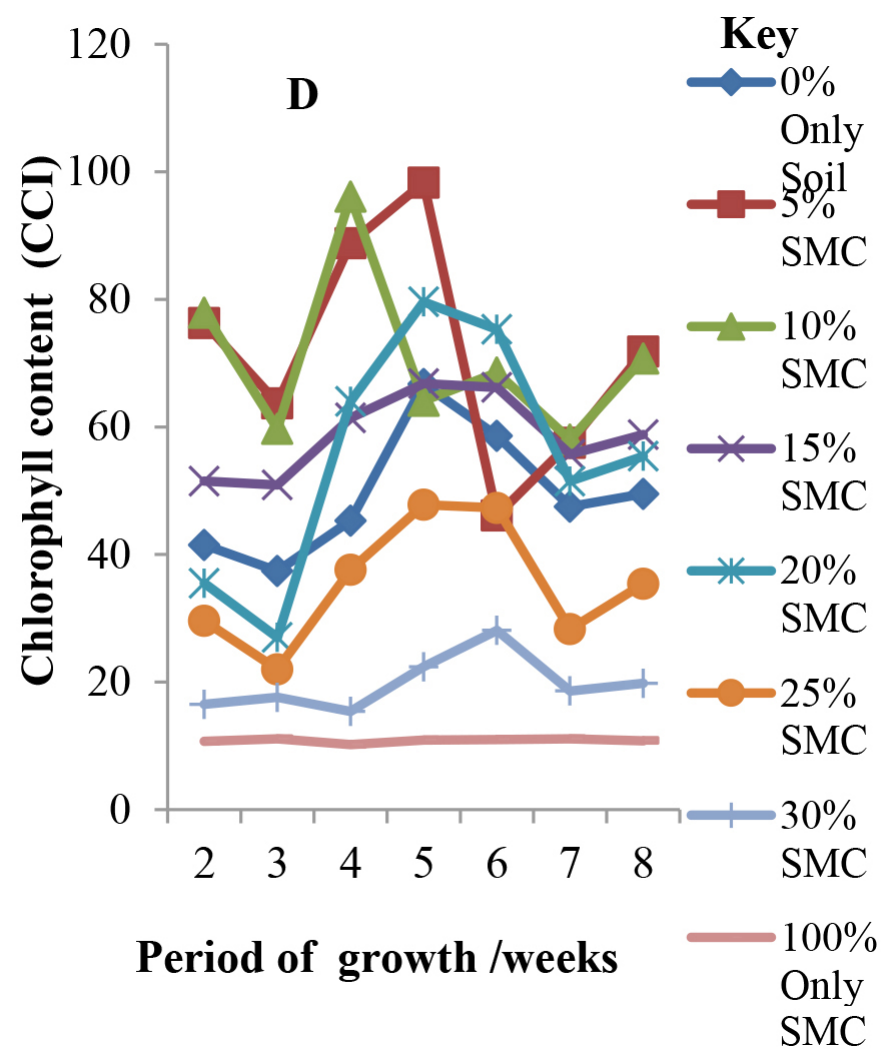
Influence of spent mushroom compost (SMC) on the growth and development of cowpea seedlings grown in varying proportions of soil: SMC under greenhouse conditions. (A) plant height; (B) leaf area; (C) number of leaves; (D) chlorophyll content.

**Figure 2:**
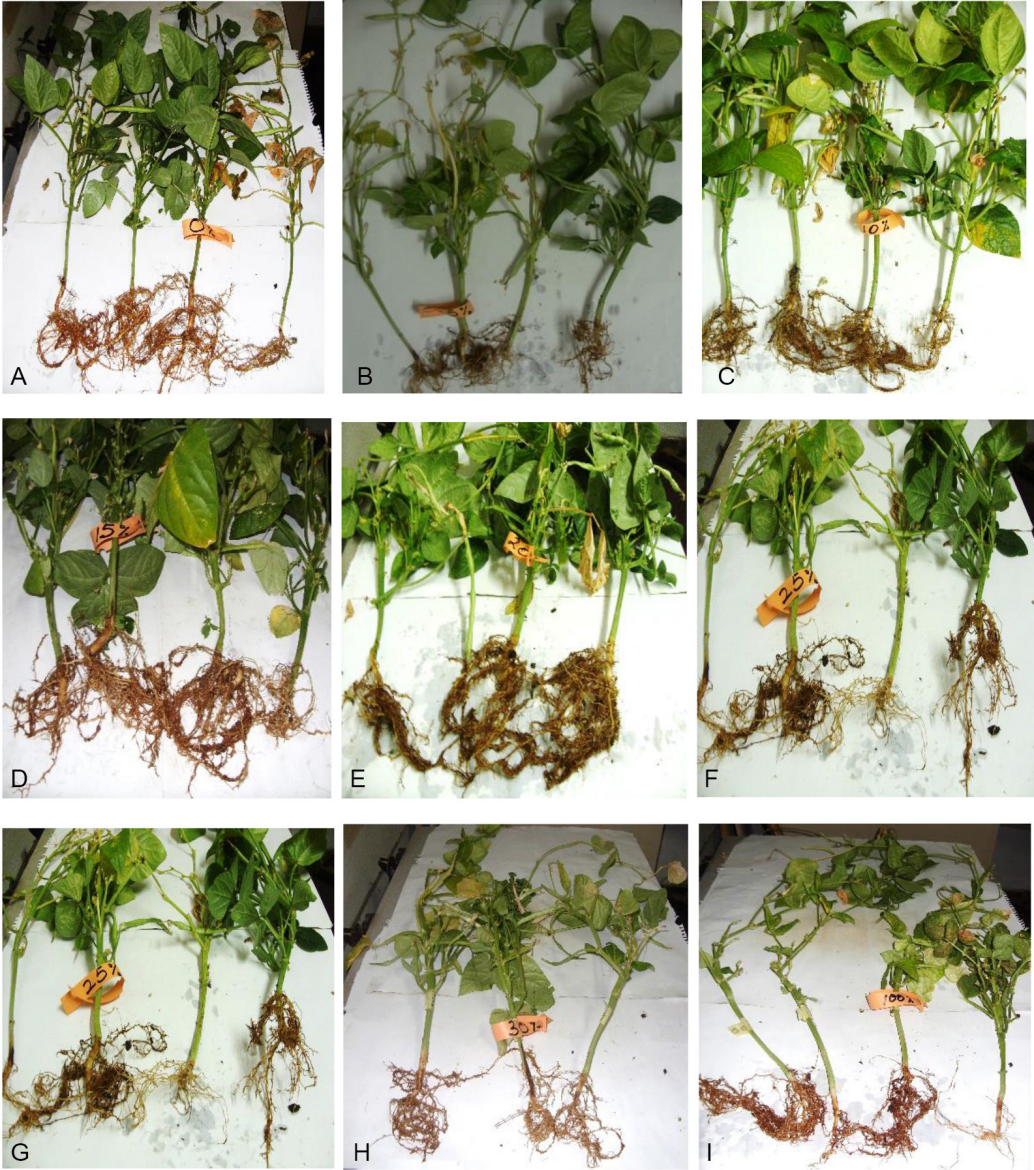
Root system of the harvested cowpea plants in the various combinations/percentages of soil: spent mushroom compost (SMC). Magnified ×1/10. *Notes*: A (0%SMC), B (5%SMC), C (10%SMC), D (15%SMC), E (20%SMC), F=G (25%SMC), H (30%SMC), I (100%SMC)

There was an initial decline in chlorophyll content after 3 weeks but was followed by an increase reaching maximum levels after 5 weeks and thereafter declined (Fig. 1D). High concentrations of 30% SMC–100% SMC reduced chlorophyll content while 5%–10% SMC recorded the highest concentration in the leaves after 4–6 weeks growth and thereafter declined (Fig. 1D). Amendment of soil with 30% SMC–100% SMC severely depressed chlorophyll formation at *p* ≤ 0.05. Fig. 2 shows vegetative growth of cowpea seedlings showing diminutive growth at 30%–100% SMC as compared to the tall luxuriant growth at 10%–25% SMC after 6 weeks at 28°C −32°C.

### Plant Height

Although plant height after 2 weeks was nearly the same, there was a clear distinction after 3–4 weeks as there was depression of growth in soil amended with 25% SMC and continued as percentage of SMC in soil increased. There was no statistical difference (p≥0.05) in plant height of seedlings cultivated in 30% SMC and 100% SMC (SMC only) and 0% (unamended soil, control). Generally, plant height (which could be correlated with dry matter accumulation) followed a sigmoid curve and the decline in plant height was visible (Figs. 1A and 2).

### Number of Leaves

This parameter estimating the efficiency of the SMC as a biofertiliser also followed a near sigmoid curve. Higher concentration of SMC depressed leaves formation and there was no statistical difference (*p* ≥ 0.05) between plants sown in 15%–100% SMC and the compost-free soil (i.e., control) (Fig. 1C).

### Total Leaf Area

Leaf area was recorded over 8 weeks period also followed a sigmoid curve. There were two categories of effect of the SMC on leaf area of seedlings. Firstly, concentrations of 5%, 10%, 15%, 20% SMC increased the leaf area (550 mm^2^–680 mm^2^; Fig. 1B). while concentration of 25% SMC and beyond depressed leaf area (300 mm^2^–400 mm^2^).

### Dry Matter Accumulation by Shoot and Root System and Fruiting Bodies

The seeds of cowpea germinated in all the pots containing different %SMC and the control. The dry matter accumulation of shoot (leaves and stems) and the root system in 12 weeks was directly commensurate with the percentage of SMC added up to 15% SMC and thereafter declined (Table 2). The highest dry weight of shoot (43.4 ± 2.0 g) and root system (0.75 ± 0.36 g) was recorded in 15% SMC and there was no statistical difference (*p* ≥ 0.05) between this and what obtained in the 10% SMC pots. The poorest growth was recorded in pots containing 100% SMC (compost only) 16.2 ± 1.18 g for the shoot and 0.21 ± 0.02 g for root system, followed by pots containing soil only (20.6 ± 1.56 g and 0.39 ± 0.14 g for shoot and root, respectively) Table 2. The trend in the growth and development parameter, height of plant, leaf area, number of leaves formed, number of axillary branches, number of flower buds, number of flowers, number of pods setting, weight of pods and seeds (data not shown) followed this general trend 5% < 10% < 15% < 20% < 25% < 30% < 100% in descending order. The differences observed were statistically significant (*p* ≤ 0.05).

### Mineral Element Composition of Soil and Spent Mushroom Compost

Table 3 summarises results obtained. Both soil and SMC did not contain iron but harboured reasonable amounts (mg/kg) of Zn, Cu, Mn, Pb, Ca, Mg, Na, P, K and N with a pH range of 6.6–6.8 (Table 3).

### Nitrogen Content of Compost and Plant During Growth

The nitrogen content of soil: compost mixtures increased with increasing percentage of the spent compost from 5%–100% (Table 4). The increase in nitrogen content of the plant during 12 weeks of growth was commensurate with the increasing percentage of the SMC in the soil (Table 4). Thus, there was transfer of nitrogen to the growing plant during the cultivation period. Percentage dry matter of the spent compost did not change significantly (*p* ≥ 0.05) in all the varying mixtures with soil (0%–30%) and was attended by corresponding dry matter accumulation of the cowpea seedlings (93.18%–93.80%) (Table 4).

### Influence of Various Combination of Soil/SMC Mixtures on *Rhizobium* and Nodulation of Cowpea and Radicle Development

It was anticipated that there will be successful development of *Rhizobium* in the soil/SMC combinations leading to successful nodulation and pod setting of the cowpea plant. This will underscore the efficacy of the SMC as a biofertiliser.

**Figure 3:**
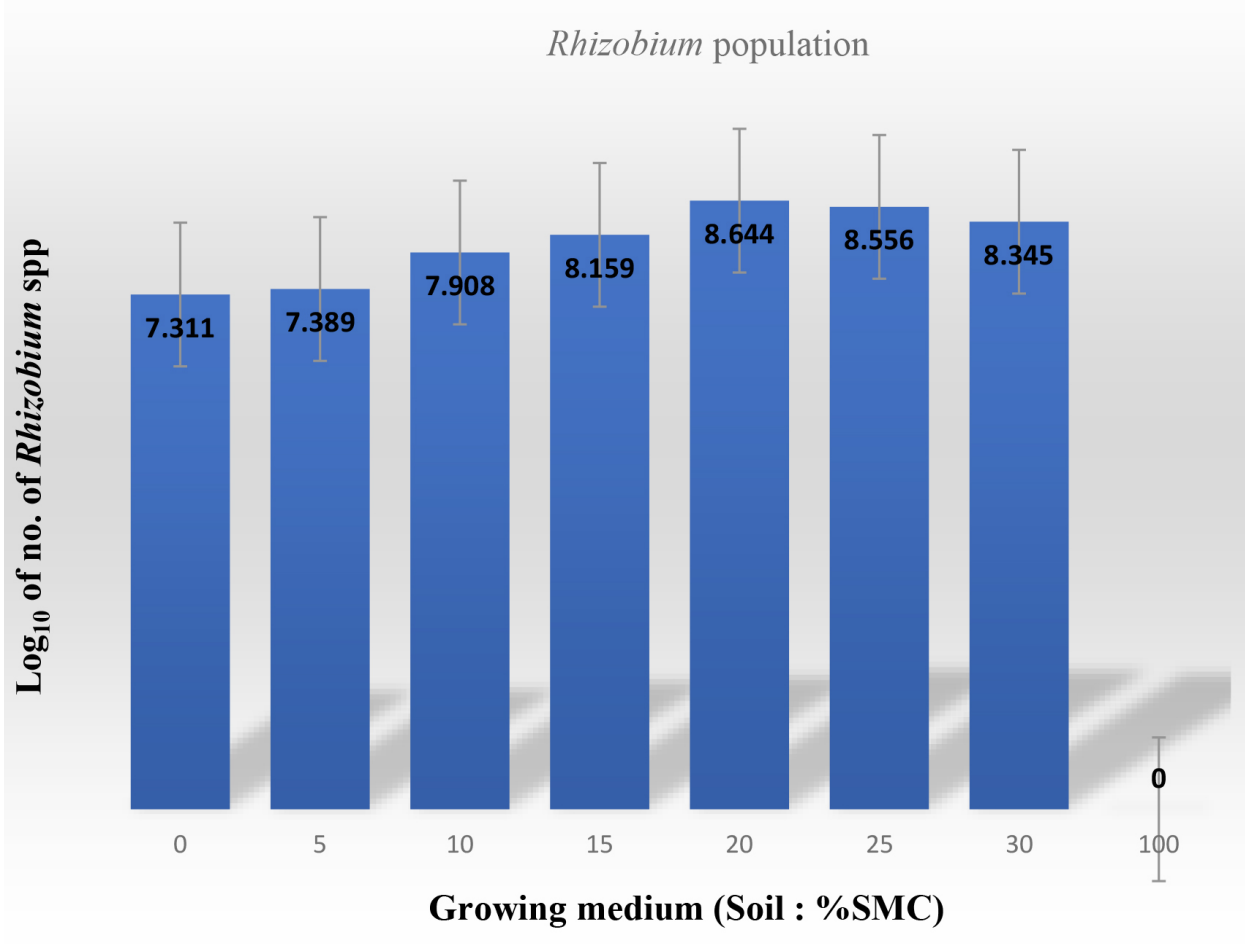
*Rhizobium* population resident in the root nodules of cowpea seedlings growing in different proportion (%) of the Soil: SMC mixtures after 12 weeks at average temperature of 28 ± 2°C.

Table 4 summarises the record of percentage germination of cowpea seed, mean length of radicles/rootlets formed, mean number of nodules, mean diameter of nodules, colour and mean number of nodules formed. Fig. 3 shows the root system of the harvested plants in the various combination percentages of soil and SMC with the attendant nodules formed. The calculated Nodule Index (a product of nodule size × nodule colour × number of nodules) are presented in Table 5.

Percentage germination of the cowpea seeds in the soil amended with 5%–30% SMC was 100% whereas in the unamended soil and SMC only 80% germination was recorded in the pots. The total weight, diameter (size) and number of nodules formed increased with increasing percentage of SMC to up to 20% SMC and thereafter declined (Table 4). The calculated Nodule Index showed that the value was highest at 5% SMC followed by 10% SMC and thereafter declined (Table 4). The colour of the inner portion of the nodules (which may indicate degree of rhizobial activity) were pink to red in nodules formed at 5%–30% SMC and white in the soil only pots while no nodules were formed in the 100% SMC (SMC only) growing medium. Fig. 3 depicts the morphology and sizes of nodules on the root system. There was a corresponding marginal increase of rhizobial population in the root nodules with increasing percentage of SMC reaching a peak of 8.32–8.57 cfu/g sample at 20%–25% SMC (Fig. 3). However, the differences were not statistically significant (*p* ≥ 0.05). No rhizobial activity population was recorded in the pots seeded with cowpea and containing only SMC medium (100% SMC).

## DISCUSSION

Lignocellulose from agricultural waste constitutes a formidable environmental waste problem in Africa not excepting Ghana. The recent aggravation of the environmental problem due to pollution from disposal of solid plastic waste into the environment has accentuated the environmental menace in Ghana. Recently, Wiafe-Kwagyan *et al*. (2017) reported in their book that rice (*Oryza sativa*) wastes can be managed in Ghana using the biotechnological cultivation of Oyster mushroom on composts formulated from rice wastes. Ghana produces an average of 541,830 metric tonnes of rice annually (Statistics and Research Information Department of Ministry of Food and Agriculture [SRID, MOFA] 2014; 2015, 2017; *Graphic Online* 2015; Millennium Development Authority [MiDA] 2012)

The government of Ghana through the “Planting for Food Initiative by the Ministry of Food and Agriculture” has increase rice cultivation extensively in almost all 16 regions in the country. The country is phasing out the import of rice by 2023. The use of rice agro-industrial wastes as potential substrates for cultivation of edible mushrooms such as *Agaricus*, *Pleurotus*, *Lentinus*, *Volvariella* species is envisaged.

Curiously, SMC are currently disposed of as waste, constitute another environmental pollution problem. However, there can be economic benefit of SMC in agriculture as bio-fertilisers, if properly harnessed.

SMC can be used as feeding material for vermicomposting, plant disease management, preparation of organic mineral biofertilisers and also for bioremediation of contaminated soils (Ahlawat *et al*. 2010). Wiafe-Kwagyan and Odamtten (2018) showed that *P. eous* cultivated on rice wastes provided favourable soil conditions as a biofertiliser for enhanced growth and yield of pepper and tomato in Ghana. Farmers from different agroecological region of Ghana are not conversant with the use of SMC as a manure or biofertiliser for soil conditioning in the cultivation of crops because of paucity of scientific data to back its use on a commercial scale.

This paper provides novel information on the use of rice lignocellulose (rice straw, rice barn and rice husk) and its supplementation to boost the production of *P. eous* and its spent compost use as biofertiliser to grow cowpea (*V. unguiculata*).

SMC mixtures with soil (5%–100% SMC) variably increased total chlorophyll content of the leaves, plant height, number of leaves per plants, leaf area and dry matter accumulation by the root and fruiting bodies (Fig. 1). Chlorophyll b was highest (20.46 mg/L–23.15 mg/L) in the 0%–5% SMC while chlorophyll a predominated 12.32 mg/L–22.57 mg/L) in the 10%–25% SMC. In the compost with 30%–100% SMC, Chlorophyll a (5.56 mg/L–9.63 mg/L) was higher than chlorophyll b (5.56–9.63mg/L). Total chlorophyll was highest at 5%–25% SMC but least at 100% SMC (17.81 mg/L) after 12 weeks (Table 1). High concentration of 30% SMC–100% SMC reduced chlorophyll content while 5%–10% gave the highest concentration of chlorophyll in the leaves. Amendment of soil with 30%–100% SMC severally depressed chlorophyll formation at *P* ≤ 0.05. This was reflected in cowpea seedling showing diminutive growth as compared to the tall luxuriant growth at 10%–25% SMC after six weeks (Fig 3). The reasons for these observed differences are not far to seek. Photosynthesis occurs in two phases; a light and dark reactions in what is termed Photosystems I and II within the chloroplasts. Reaction sites for light and dark reactions could have been influenced by the levels of mineral elements and nutrients and chlorophyll a and chlorophyll b. Recent data of Wiafe-Kwagyan (2014) showed that the SMC of *P. eous* contained 90.8 ± 1.79% dry matter: 50.42 ± 4.64% ADF. Calcium, potassium, nitrogen, sodium and some heavy metals such as copper, iron, manganese, lead and zinc present at pH 6–6.8 could have influenced the development of the cowpea plant either positively or negatively (Table 3).

The plant height of the cowpea seedlings under the soil SMC mixture regimes (0%–100%) were initially nearly the same, but there was a clear distinction after 3–4 weeks attended by a depression in growth in soil amended with 25% SMC and continued to decline with increased percentage of SMC. Generally, plant height records followed a sigmoid curve and the decline in plant height was visible (Figs. 1A and 2). According to Guo and Chorover (2004), high concentration of mineral elements may be inhibiting and may limit growth beyond a certain threshold concentration such that beyond 15% SMC there was a reduction in shoot and root weight of the cowpea (Table 2).

The effect of the SMC/Soil mixing serving as a biofertiliser also followed a near sigmoid curve (Fig. 1C), so far as the number of leaves formed was concerned. Higher concentration of SMC depressed leaf formation ranging from 5–22 in eight weeks (Fig. 1C) while there was no statistical difference (*P* ≥ 0.05) between plants sown in 15%–100% SMC and the compost free soil (i.e., control medium) (Fig. 1C). It seems the reduction in leaf number was less severe but there were two categories of the effect of the SMC on the leaf area of the cowpea seedlings. Firstly, concentrations of 5%, 10%, 15%, 20% SMC increased leaf area (550 mm^2^–680 mm^2^) in conformity with their photosynthetic efficiency while concentrations of 25% and beyond depressed leaf area (200 mm^2^–400 mm^2^). There is therefore a direct proportionate relationship between number of leaves formed; leaf area and photosynthetic capacity (total chlorophyll).

It was anticipated that the differential influence of the varying percentages of SMC would reflect in the dry weight accumulation of the seedling. Data from this paper support this viewpoint. Although all the seeds of cowpea germinated in all the pots containing different % SMC’s and the control, the dry matter accumulation of the shoot (leaves and stem) and the root system in 12 weeks was directly commemorate with the % SMC added up to 15% SMC and thereafter declined (Table 2). For example, the highest dry weight of shoot (43.4 ± 2.0 g) and root system (0.75 ± 0.26 g) was recorded in the 15% SMC pots and there was no statistical difference (*P* ≥ 0.05) between this value and what obtained in the 10% SMC pots. Correspondingly, the poorest growth was recorded in the pots containing compost only (16.2 ± 1.18 g for the shoot and 0.21 ± 0.02 g for the root system). Pots containing soil only recorded 20.6 ± 1.56 g for the shoot system and 0.39 ± 0.14 g for the root system (Table 2). Clearly, the trend in the growth and development parameters used in this study (height of plant, leaf area, number of leaves, dry weight of shoot and roots formed not excepting number of axillary branches, number of flower buds, flowers, pods setting, weight of pods, weight of seeds (Data not shown) followed a trend which were statistically significant (*P* ≤ 0.05) as follows; 5% < 10% < 15% < 20% < 25% < 30% < 100% (in decreasing order).

According to Guo and Chorover (2004), high concentrations of mineral elements (such as obtained in increasing % SMC) may limit or inhibit growth of the cowpea beyond a certain threshold. This agrees with our findings. Increasing % SMC beyond 15% drastically reduced the listed parameters used to access growth especially the root and shoot weight of the cowpea plant in this present study.

There was another interesting observation. The cowpea plant seems to efficiently assimilate nitrogen. The nitrogen content of the soil; compost mixture increased with increasing percentage of the spent compost from 5%–100% SMC (Table 4). This increase was commensurate with the increasing percentage of the SMC in the soil (Table 4). Thus, there was a transfer of nitrogen to the growing plant during the growth period of 12 weeks. The dry matter of the spent compost did not change significantly in all the varying mixture with soil (0%–30%) and this was attended by a corresponding dry matter accumulation of the cowpea seedlings (93.18%–93.80%) (Table 4). Biological activity of nitrogen fixation is related to the dry matter yield (Antoun & Prévost 2005; Antoun *et al*. 1998).

It was conjectured that the growing environment of the soil: SMC mixture will successfully support development of *Rhizobium* leading to successful nodulation and pod setting. This will underscore the efficiency of the SMC as a biofertiliser and supporter of rhizobial root nodulation leading to nitrogen fixation. There were good seed germination regimes ranging from 80%–100% in the soil amended with 5%–30% SMC as well as in the unamended soil and SMC only (Table 5) with a corresponding vegetative growth.

Legumes, like cowpea are able to form symbiotic relationship with nitrogen – fixing soil bacteria collective called rhizobia (belonging to the genera *Rhizobium*, *Bradyrrhizobium* and *Azorhizobium*) which elicit the formation of nodules on legume roots (and stem of some species) of specific organs; the nodules in which they fix nitrogen (Denarie *et al*. 1993; Antoun *et al*. 1998; Brewin 2010; Clúa *et al*. 2018; Mendoza-Suarez *et al*. 2020).

Nodule formation in this paper was highest in 5% SMC: soil mixture (89) and this declined with increasing proportion of SMC: soil mixture (5%–30% SMC) (Table 5) but completely depressed nodule formation by cowpea growing in SMC only (100% SMC). This implies that the bacterial rhizobial population came from the soil and was stimulated by requisite nutrients from the spent mushroom compost, SMC (Table 5, Fig. 3). This is the first record of SMC in mixture with soil stimulating and enhancing root nodule formation by cowpea at 5% SMC up to 30% SMC (Table 5) but failing to nodulate in the absence of soil microbiota.

Using the criteria of Nodule Index (Rebah *et al*. 2002), the best nodule size and weight were formed in the in the growing medium of 5% SMC and 10% SMC (i.e., 18 and 12, respectively (Table 5). The nodules were pink-red in colour epitomising leghaemoglobin, viable to initiate nitrogen fixation in the soil.

The SMC of *P. eous* P-31 at low percentage mixture with soil (5%–30% SMC) can therefore improve nodulation of cowpea in soil and enhance pod formation. The economic and ecological importance of legumes is evidenced by high number of species that are cultivated and commercialised, as well as their ability to obtain nitrogen from a symbiotic interaction with soil bacteria known as rhizobia (Clúa *et al*. 2018).

The family of flowering plant of Fabaceae includes species of agronomic importance such as common bean (*Phaseolus vulgaris*), alfalfa (*Medicago sativa*), soybean (*Glycine max)*, pea (*Pisum sativum*), lentil (*Lens culinaris*) and black eye beans (*V. unguiculata*). These present findings extend the list of plants whose unique capacity to establish a nitrogen fixing symbiosis can be enhanced by using SMC of *P. eous* to increase nodulation and nitrogen fixation in the commercial production in the near future. Nitrogen fertilisation is extremely expensive and generates ecological risks such as water eutrophication and emission of atmospheric greenhouse gases that contribute to global warming (Clúa *et al*. 2018). Biological nitrogen fixation on the other hand is an ecologically friendly alternative. However, it is restricted to the symbiotic interaction of a small group of economically important plants (mainly legumes and actinorhizal plants) with nitrogen-fixing microorganisms. This paper has demonstrated the value of SMC of *P. eous* to boost nitrogen fixation by *V. unguiculata* and opens the way to try its usefulness on other legumes as stated above.

Wiafe-Kwagyan *et al*. (2017) showed that rice (*Oryza sativa*) wastes can be managed in Ghana using the biotechnological cultivation of Oyster mushroom (*Pleurotus* spp.) in a compost formulated from rice wastes (Wiafe-Kwagyan *et al*. 2016). The resultant SMC of *P. eous* has been shown to provide favourable soil conditions for cultivating fruits, vegetables, such as tomato and pepper in Ghana (Wiafe-Kwagyan & Odamtten 2018) and for cowpea nodulation and vegetative growth in this article.

## CONCLUSION

The greenhouse studies using varying percentages of soil with SMC from rice waste (0%–100% SMC) used in cultivating *P. eous* strain P-31 has shown that the effect of the compost was different depending on the proportion of the mixture of SMC and soil. Low percentage of the soil compost mixture 5%–25% SMC variably improved plant height, leaf area, number of leaves, total chlorophyll of cowpea leaves however SMC concentration beyond 30% depressed growth. rhizobia population and nodulation was increased by 5%–25% SMC and declined with increased percentage to the plant that Rhizobia were not found in the pots containing unamended SMC (i.e., 100% SMC, without soil). The highest Nodule Index was at 5% SMC (18 plants) followed by 10% SMC (12 plants) and thereafter declined to zero Nodule Index at 30% SMC. There are therefore future prospects for practical application of these findings with anticipated beneficial economic outcomes.

## Figures and Tables

**Table 1:** Influence of spent mushroom compost (SMC) on chlorophyll content of leaf extract of cowpea seedlings.

Treatment (%SMC)	Chlorophyll a (mg/L)	Chlorophyll b (mg/L)	Total chlorophyll (mg/L)
0	9.65	20.46	30.89
5	9.33	23.15	33.33
10	12.32	6.75	19.43
15	20.38	10.64	31.59
20	18.65	9.03	28.18
25	22.57	12.30	35.46
30	18.30	9.63	28.43
100	11.95	5.56	17.81

**Table 2:** Dry matter accumulation by cowpea seedlings after 12 weeks of growth in pots containing indicated percentage of soil: SMC mixtures in the greenhouse at 28°C–32°C.

Substrate treatment (%SMC)	Mean dry weight (g ± SE) of	

	Shoot system	Root system
0 (Soil only)	20.6 ± 1.58^a^	0.39 ± 0.14^a^
5	24.2 ± 1.58^a^	0.50 ± 0.16^b^
10	42.7 ± 1.50^b^	0.71 ± 0.32^c^
15	43.4 ± 2.00^b^	0.75 ± 0.36^c^
20	36.6 ± 2.76^c^	0.41 ± 0.20^d^
25	34.4 ± 4.29^c^	0.33 ± 0.11^a^
30	22.9 ± 0.11^a^	0.30 ± 0.05^a^
100	16.2 ± 1.18^d^	0.21 ± 0.02^e^

**Table 3:** Mineral composition of soil and spent compost used as growing medium for cowpea seedlings.

Physical and chemical properties	Growing medium	

Mineral concentration (mg/kg)	Soil	Spent mushroom compost of *P. eous* strain-P-31
Zn	0.031 ± 0.0011	0.017 ± 0.001
Cu	0.000 ± 0.00	0.005 ± 0.0001
Mn	0.269 ± 0.0013	0.069 ± 0.001
Pb	0.129 ± 0.001	0.013 ± 0.001
Ca	0.471 ± 0.003	0.633 ± 0.003
Mg	0.444 ± 0.002	0.893 ± 0.003
Fe	0.000 ± 0.00	0.000 ± 0.00
Na	0.217 ± 0.001	0.196 ± 0.002
P	0.890 ± 0.015	0.795 ± 0.004
K	0.118 ± 0.0001	0.281 ± 0.0013
N	0.819 ± 0.013	0.978 ± 0.0053
% dry matter	0.98 ± 0.006	10.68 ± 2.030
pH	5.80 ± 1.520	6.60 ± 1.870

**Table 4:** Correlation between nitrogen content of growing medium and assimilation of nitrogen by cowpea seedlings cultivated in the indicated proportions of spent mushroom compost with soil at 28°C –32°C for 12 weeks.

Type of treatment (%SMC) mixture with soil	Total (%) Nitrogen content			Moisture content (%)		

	% dry matter of growing media	% dry matter of plant sample	Compost (%)	Plant (%)	Growing media	Plant
0 (Soil only)	98.68	93.76	0.07	1.55	3.15	83.37
5	98.59	93.27	0.16	1.62	5.87	87.13
10	98.41	93.20	0.19	1.92	7.46	87.59
15	98.48	93.80	0.25	2.18	8.83	87.90
20	98.40	93.43	0.30	2.34	11.04	87.45
25	98.03	93.74	0.43	2.25	12.54	87.30
30	97.99	93.18	0.40	2.08	20.63	88.20
100 (SMC only)	94.10	94.18	1.16	1.60	44.71	89.34

**Table 5:** Estimation of Nodule Index of nodules formed by cowpea plant grown in varying soil: compost mixtures under greenhouse condition at 28 ± 2°C for 9 weeks.

Growing medium (%SMC) (Compost: Soil mixture)	Nodule size (mm) nodule index	Nodule weight (mg) nodule index
0	2	4
5	18	18
10	12	12
15	8	8
20	8	8
25	4	4
30	4	2
100	0	0

*Notes*: Nodule Index = A × B × C ≤ 18 (after Rehab *et al*. 2002)
